# Smart Poly(N-isopropylacrylamide)-Based Hydrogels: A Tour D’horizon of Biomedical Applications

**DOI:** 10.3390/gels11030207

**Published:** 2025-03-15

**Authors:** Soumya Narayana, B. H. Jaswanth Gowda, Umme Hani, Mohammed Gulzar Ahmed, Zahrah Ali Asiri, Karthika Paul

**Affiliations:** 1 Department of Pharmaceutics, Yenepoya Pharmacy College & Research Centre, Yenepoya (Deemed to be University), Mangalore 575018, Karnataka, India; kalikollur123@gmail.com; 2 Department of Pharmaceutics, College of Pharmacy, King Khalid University, Abha 61421, Saudi Arabia; uahmed@kku.edu.sa (U.H.); z.asiri@kku.edu.sa (Z.A.A.); 3 Department of Pharmaceutical Chemistry, JSS College of Pharmacy, JSS Academy of Higher Education and Research (JSSAHER), Mysuru 570015, Karnataka, India; karthikananjundan17@gmail.com

**Keywords:** hydrogel, PNIPAM, thermoresponsive, drug delivery, wound healing, tissue engineering

## Abstract

Hydrogels are innovative materials characterized by a water-swollen, crosslinked polymeric network capable of retaining substantial amounts of water while maintaining structural integrity. Their unique ability to swell or contract in response to environmental stimuli makes them integral to biomedical applications, including drug delivery, tissue engineering, and wound healing. Among these, “smart” hydrogels, sensitive to stimuli such as pH, temperature, and light, showcase reversible transitions between liquid and semi-solid states. Thermoresponsive hydrogels, exemplified by poly(N-isopropylacrylamide) (PNIPAM), are particularly notable for their sensitivity to temperature changes, transitioning near their lower critical solution temperature (LCST) of approximately 32 °C in water. Structurally, PNIPAM-based hydrogels (PNIPAM-HYDs) are chemically versatile, allowing for modifications that enhance biocompatibility and functional adaptability. These properties enable their application in diverse therapeutic areas such as cancer therapy, phototherapy, wound healing, and tissue engineering. In this review, the unique properties and behavior of smart PNIPAM are explored, with an emphasis on diverse synthesis methods and a brief note on biocompatibility. Furthermore, the structural and functional modifications of PNIPAM-HYDs are detailed, along with their biomedical applications in cancer therapy, phototherapy, wound healing, tissue engineering, skin conditions, ocular diseases, etc. Various delivery routes and patents highlighting therapeutic advancements are also examined. Finally, the future prospects of PNIPAM-HYDs remain promising, with ongoing research focused on enhancing their stability, responsiveness, and clinical applicability. Their continued development is expected to revolutionize biomedical technologies, paving the way for more efficient and targeted therapeutic solutions.

## 1. Introduction

Gels are distinctive materials positioned between the states of solids and liquids, formed by crosslinked networks of colloidal polymers [1]. These structures exhibit a remarkable ability to undergo volumetric changes, either expanding (swelling) or contracting (shrinking), in response to their interaction with a suitable solvent [2]. The mechanism behind this behavior involves a dynamic balance of opposing forces acting within the gel matrix. Swelling is initiated when repulsive forces (such as those generated by osmotic pressure and electrostatic repulsion) surpass attractive interactions like hydrophobic and hydrophilic affinities [3,4]. On the other hand, shrinkage occurs when attractive forces, including hydrogen bonds, dispersion forces, and van der Waals interactions, dominate over the repulsive forces. This delicate equilibrium between attraction and repulsion underpins the adaptive nature of gels and their responsiveness to environmental stimuli [5].

Hydrogels have emerged as critical components in modern medicine, significantly enhancing applications in drug delivery systems, as well as advancing tissue engineering and the development of 3D-printed artificial organs [6,7,8]. Hydrogels are characterized by a water-swollen, crosslinked polymeric network structure that exhibits an extraordinary ability to absorb and retain substantial amounts of water (up to 100 times their weight) while resisting dissolution [9,10,11]. This combination of water retention and structural stability makes hydrogels highly versatile and indispensable for a wide range of biomedical applications. Hydrogels that respond to environmental triggers, often labeled as “smart” or “adaptive” hydrogels, belong to a specialized class known for their remarkable ability to swell and form gels [12,13,14,15]. These advanced materials exhibit sensitivity to changes such as pH levels, temperature, light exposure, electric fields, and ionic concentrations, adjusting their volume or transitioning between liquid (sol) and semi-solid (gel) states in a reversible manner [16,17,18,19,20].

Thermoresponsive polymers demonstrate gel formation below their lower critical solution temperature (LCST) [21,22]. Positive thermosensitive hydrogels contract when cooled below the LCST and dissolve at temperatures above the upper critical solution temperature (UCST), with chemically crosslinked types swelling more significantly at lower temperatures [23,24]. On the other hand, negative thermosensitive hydrogels form a gel from a sol as the temperature surpasses the LCST, making them suitable for applications like the controlled release of therapeutics [25]. The discovery of poly(N-isopropylacrylamide)’s (PNIPAM’s) thermal phase transition properties by Scarpa et al. in 1967 marked a pivotal moment in the evolution of thermoresponsive polymer systems. Initially synthesized in 1956, PNIPAM’s phase behavior in aqueous solutions was first charted in 1968 [26]. Later, in 1986, Pelton and Chibante introduced thermoresponsive microgels crafted from PNIPAM, propelling its prominence in smart material applications [27,28,29]. Structurally, PNIPAM features amide and isopropyl groups that confer an LCST of approximately 32 °C in water [30,31]. Hydrogels derived from crosslinked PNIPAM, or its variants, display a remarkable ability to undergo reversible volume changes, transitioning between swollen and shrunken states near the LCST. Beyond these volume changes, the phase transition also influences characteristics such as wettability, optical clarity, and dielectric properties [32,33,34].

This review focuses on the unique properties and behavior of smart PNIPAM and explores its diverse synthesis methods. A detailed assessment of its biocompatibility is provided, along with an extensive analysis of the structural and functional modifications applied to PNIPAM-based hydrogels (PNIPAM-HYDs). The discussion then transitions to the biomedical applications of PNIPAM, highlighting its utility in cancer therapy, phototherapy, wound healing, TE, ocular conditions, skin diseases, and many more. Furthermore, this review elaborates on the various delivery routes, followed by an in-depth examination of relevant patents that emphasize the innovative approaches underlying their development and therapeutic potential.

## 2. Smart Characteristics of PNIPAM

PNIPAM has garnered significant interest over recent decades because it is a thermoresponsive polymer known for its unique behavior of increasing solubility at lower temperatures, which leads to a volume phase transition through hydrogen bonding [35]. This is a synthetic polymer, derived from the acrylamide monomer NIPAM, and its structure contains both hydrophobic isopropyl side groups and hydrophilic amide groups. In aqueous environments, PNIPAM undergoes a phase change, shifting from a hydrophilic to a hydrophobic state when heated above its LCST. Below the LCST, the hydrogel can absorb water and swell. PNIPAM can be modified through copolymerization to develop stimuli (temperature)-sensitive hydrogels [33,36]. The stimuli-sensitive behavior of PNIPAM arises from the interactions between its functional groups and solvent molecules. When PNIPAM converts from a sol to a gel at the LCST, it is particularly suitable for wound dressings, TE scaffolds, and drug delivery techniques [37]. The swelling and shrinking characteristics of PNIPAM-based smart hydrogels can be easily regulated by copolymerizing them with more hydrophilic or hydrophobic monomers. Notably, these hydrogels can undergo property changes triggered by various methods, such as direct and indirect heating. This versatility allows them to respond to temperature, light, electric fields, and magnetic fields [38,39]. The unique features of PNIPAM-based smart hydrogels enable them to perform functions like living cells and other intelligent biological systems, changing shape, self-regulating, and rupturing. For instance, their volume change capabilities allow these hydrogels to control material flow—swelling can block channels, while shrinking can open them. This property is crucial for several biomedical applications [40,41,42].

## 3. Synthesis of PNIPAM-HYDs

PNIPAM-HYDs are typically synthesized using three main methods: free radical polymerization (FRP), controlled living radical polymerization (CLRP), and graft copolymerization (GCoP). Among these, FRP and CLRP are the most employed techniques for developing PNIPAM-HYDs [43,44].

### 3.1. Free Radical Polymerization

The process entails polymerizing the readily available hydrophilic NIPAM monomer along with small quantities of crosslinking agents. Ammonium persulfate (APS) is used as an initiator and is broken down into free radicals, either by heating or UV irradiation. These free radicals react with the unsaturated C=C bonds of the monomers, causing the chain to grow until termination occurs, which happens when two free radical molecules combine [45]. A catalyst is employed to speed up radical polymerization. This method has several benefits such as the ability to easily control reaction conditions, the flexibility to use a diverse range of monomers, and the option to work with both aqueous and organic solvents. On the other hand, some drawbacks include challenges in controlling the size and dispersion of the particles, the degree of polymerization, etc. [35]. Strachota et al. developed a PNIPAM clay-based hydrogel using APS as the initiator and TEMED as the accelerator. The results disclosed that the process of gel formation was improved based on the findings from these experiments [46,47]. The different initiator classes for FRP include peroxy compounds, which are used to initiate vinyl polymerization. Under the azo compound class, triphenyl azobenzene was first used as a polymerization initiator by Bickmann and his team [48].

### 3.2. Controlled Living Radical Polymerization

The LRP method is characterized by rapid initiation, slow propagation, and minimal termination. Atom transfer radical polymerization (ATRP) is a form of CLRP that allows for the precise management of reaction kinetics, molecular weight distribution (resulting in low dispersion), and the design of complex molecular architectures. ATRP is a synthetic macromolecular technique that uses CLRP to synthesize well-defined polymers. It has played a key role in the development of advanced materials, such as bioconjugates and nanocomposites [49]. ATRP can be performed in both organic solvents and aqueous environments for NIPAM, though it can face challenges when used for acrylamides. Research by Matyjaszewski et al. has demonstrated that certain functional (metha) acrylamides can be successfully polymerized using ATRP. However, it is widely accepted that the polymerization of NIPAM is not easily controlled through ATRP. Instead, it can be more effectively controlled using reversible addition–fragmentation chain transfer (RAFT) polymerization [35,50].

### 3.3. Graft Copolymerization

Graft copolymerization is a process where one or more distinct monomers are attached to the backbone of an existing polymer, forming side chains. These monomers differ chemically from those in the original polymer. The resulting graft copolymer exhibits unique characteristics both in structure and composition compared to the parent polymer. This reaction allows for the incorporation of diverse functional groups into the polymer. As a result, graft copolymers can have tailored properties for specific applications [51]. Hydrogels usually exhibit random particle size distributions, whereas emulsion polymerization enables the control of particle size from micro- to nanoscale. The literature reported a method for activating the surface of polypropylene (PP) meshes using oxygen plasma, followed by the covalent grafting of a PNIPAM-co-MBA-HYD onto this surface. The substrate is immersed in a solution with a NIPAM monomer, MBA crosslinker, and peroxide activator (APS) to initiate radical polymerization. This leads to covalent bond formation between the hydrogel and the activated PP fibers, promoting additional growth. Lastly, the PP–gel bilayer is taken out of the reaction mixture, rinsed, and neutralized to a pH of 7 to improve the gel grafting efficiency. This bilayer system converts the inert PP surface into a temperature-responsive implant that can adapt to changes in local temperature and humidity [35,51]. 

## 4. Biocompatibility Status of PNIPAM

PNIPAM is a widely studied temperature-sensitive polymer known for its unique ability to switch from a sol to a gel state. This property makes PNIPAM an excellent candidate for various biomedical applications. In drug delivery, PNIPAM can easily incorporate drugs by dissolving them in the solution at lower temperatures and then raising the temperature above its LCST to form a gel [52,53]. This allows for precise control over targeted site drug release. In tissue engineering, PNIPAM can be injected as a solution that transforms into a 3D hydrogel at body temperature, facilitating tissue repair while supporting cell viability and growth. For wound dressings, the temperature-sensitive phase conversion of PNIPAM helps regulate how the gel adheres to and detaches from the skin. Additionally, PNIPAM offers tunable structures and low toxicity, enhancing its suitability for biomedical uses [54,55]. PNIPAM degrades into smaller molecules like NIPAM, cyclic imides, diisopropylamine, and dimer/trimer fragments. This breakdown happens through chain scission, typically under high temperatures or certain chemical conditions. Other degradation products may include peptide fragments or remnants of crosslinking agents, depending on the specific degradation method [56]. The literature reports that an indirect cytotoxicity test revealed that this copolymer has relatively low cytotoxicity, based on observations with 3T3 fibroblast cells. These findings suggest that it is minimally toxic to the cells. Consequently, the copolymer demonstrates good biocompatibility [57].

Despite their appealing advantages, PNIPAM-HYDs have notable limitations, including low biodegradability, inadequate mechanical strength, limited drug loading capacity, and their tendency towards the burst release of drugs. These issues significantly restrict their practical applications. To address these shortcomings, researchers have focused on enhancing the performance of PNIPAM-HYDs. A promising approach involves creating PNIPAM-HYDs by integrating them with suitable systems, for example, inorganic nanoparticles (NPs), organic self-assemblies, etc. [58]. These composite hydrogels offer improved or additional features, including better biocompatibility, increased mechanical strength, drug loading capabilities, and the targeted and controlled release of drugs. These improvements are expected to expand the range of biomedical applications for PNIPAM-HYDs [59]. A variety of methods have been used to produce PNIPAM-HYD composites, including copolymerization with various monomers, the integration of polymeric NPs, and the creation of interpenetrating polymer networks (IPNs) or nanocomposite hydrogels. Key factors, such as temperature, pH conditions, the degree of crosslinking, ionic strength, etc., can be manipulated to improve the mechanical properties of these hydrogels [59]. IPNs consist of multiple natural or synthetic polymers that are interlinked at the molecular level, making them difficult to separate without breaking chemical bonds. Semi-IPNs involve linear polymer chains that penetrate a crosslinked network, while full IPNs have both networks crosslinked. The enhanced mechanical strength of IPN hydrogels is primarily achieved through the polymerization of natural hydrophilic polymers in combination with synthetic polymers and natural proteins [34,54]. Double-network (DN) hydrogels represent an innovative type of interpenetrating network hydrogel known for their exceptional mechanical characteristics, including tensile strength ranging from 1 to 10 MPa and compressive fracture stress between 20 and 60 MPa. Additionally, they possess enhanced moisture absorption capabilities, making them suitable for use as wound dressings [54].

Recent studies have indicated that PNIPAM-IPN-HYD exhibits enhanced mechanical properties [60]. Examples include chitosan (CS)–PNIPAM-IPN-HYD, PAA/PNIPAM-HYD [61], and luteolin–hyaluronic acid (HA)–PNIPAM-HYD [62]. Another approach to enhancing the mechanical properties of PNIPAM-HYD is through copolymerization with optical or chemical crosslinkers, using an initiator within a solvent system. Recent research has investigated various PNIPAM-based copolymeric hydrogel systems, such as Laponite^®^ platelets–PNIPAM-HYD, polypyrrole–PNIPAM-HYD, polyethylene glycol diacrylate (PEGDA)–CSCS–PNIPAM-HYD [63], and CS–N-2-hydroxypropyl trimethylammonium chloride (HACC)–PNIPAM-HYD, demonstrating improvements in both biological and mechanical properties [64]. Recently, NP-loaded composite HYDs have garnered significant interest due to their high responsiveness to stimuli, reversible deformation, simple one-pot synthesis, and impressive biological and mechanical characteristics, such as increased elongation strength and enhanced compression resistance. These hydrogels can be created through physical crosslinking or covalent bonding. Incorporating inorganic and metallic NPs, such as ceramics, hydroxyapatite, and carbon-based materials, into PNIPAM-HYD systems can enhance their biological properties and mechanical strength, making them ideal for TE and wound healing applications [54,65].

## 5. Modifications of PNIPAM-HYD

Hydrogels have gained significant interest recently due to their unique properties, including high water content, softness, flexibility, and biocompatibility [66]. These hydrogels are polymeric networks formed through either the chemical or physical bonding of water-soluble polymers [67,68]. Reversible or physical gels are held by non-covalent bonds or interactions between polymer chains, while permanent or chemical hydrogels are formed through chemical crosslinking [69,70]. Thermoresponsive behavior, particularly the LCST, can be readily adjusted by altering the balance between hydrophobic and hydrophilic components. Additionally, changes in the polymer’s molecular weight and structure can also influence this behavior. Grafting density has been shown to affect the thermoresponsive properties of polymers. PNIPAM is the most extensively studied polymer in relation to grafting effects, as it undergoes a conformational change above its LCST. Both the length and density of grafts have been found to influence its thermoresponsive behavior and morphology [71,72]. PNIPAM has been widely used to develop thermosensitive nanoparticles for different applications. These nanoparticles are synthesized through several techniques, including emulsion polymerization, photoemulsion polymerization, ATRP, FRP, and in situ chemical crosslinking. Each method is suitable for various fields, including drug delivery and sensors [28].

### 5.1. Physical Crosslinking

Various physical crosslinking techniques, such as ionic interactions, hydrophobic bonds, and protein interactions, can enhance the toughness and self-healing properties of hydrogel systems, which are crucial for their use in biomedical applications [34]. Hydrogels formed through ionic interactions, such as the dynamic interaction of metal–ligand bonds, exhibit good biological activity. However, several drawbacks such as weak mechanical properties restrict the practical applications of these techniques. Hydrogen bond interactions represent another form of physical crosslinking. Although these bonds are generally unstable in aqueous conditions, they can be reformed after disruption. This method has proven effective in preparing IPN hydrogels for application in wound healing dressings. Another widely used physical crosslinking technique is freeze–thaw, which involves the formation of ice crystals that encase the polymeric chains. This process is followed by the creation of a microporous structure as the crystals melt [12,13,14,15].

### 5.2. Chemical Crosslinking

Research has shown that chemical crosslinking PNIPAM with materials like elastin-like proteins, HA, alginate, monoacryloxyethyl phosphate, and others can enhance the hydrogel’s physical properties [73,74].

#### 5.2.1. PNIPAM Crosslinking Through Elastin-Like Polypeptides

Elastin-like polypeptides (ELPs) are synthetic, biomimetic protein polymers modeled after the repeating hydrophobic motifs found in tropoelastin. Their diverse applications in drug delivery and tissue engineering have garnered significant interest [75]. One study reported that temperature-sensitive hydrogels incorporating ELPs exhibit temperature variations between 29 °C and 37 °C. PNIPAM-based scaffolds have also been utilized for creating cell sheets and have been modified. Cell attachment is typically achieved by adding adhesive molecules to modify the surface. Among available cell culture systems, dynamic systems are the most advanced for bone tissue engineering due to their efficient nutrient delivery and waste removal. The flow perfusion bioreactor system is particularly significant for bone tissue engineering, as it provides mechanical stimulation and mitigates perfusion limitations. In this study, increased cell proliferation was linked to the use of dynamic conditions. The incorporation of ELPs into PNIPAM films led to improved initial cell attachment compared to PNIPAM films alone, emphasizing the significance of cell-adhesive molecules. The PNIPAM scaffold with adsorbed ELPs demonstrated better cell attachment on day one compared to scaffolds without ELPs [76].

#### 5.2.2. PNIPAM Crosslinking Through Alginate

**Figure 1:**
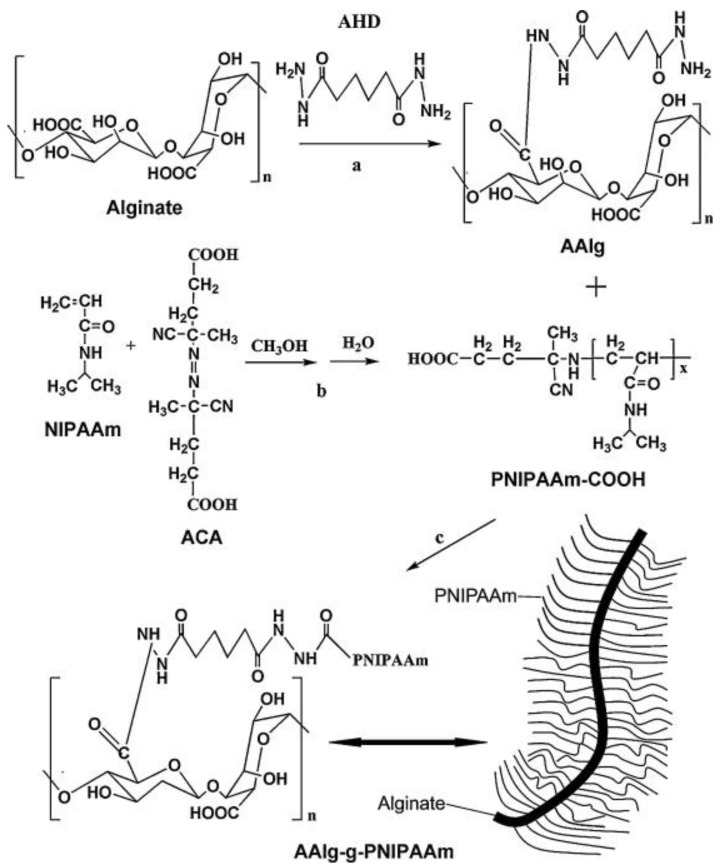
Synthetic route and molecular structures of AAlg (a), PNIPAAm (b), and comb-like AAlg-g-PNIPAAm (c), Reprinted with permission from [78], copyright 2012, Elsevier.

Alginate, a naturally occurring anionic polymer from brown seaweed, is widely used in biomedical applications due to its biocompatibility, low toxicity, and cost-effectiveness [77]. In 2012, Tan et al. explored biodegradable and thermosensitive comb-like hydrogel copolymers for TE applications. These copolymers were synthesized by combining carboxylic end-capped PNIPAM (PNIPAM-COOH) with aminated alginate via amide bond linkages. This unique structure, with alginate as the hydrophilic backbone, resulted in an increased LCST and swelling ratios, with the LCST around 35 °C (Figure 1). Consequently, this property facilitates gel formation at body temperature. The study also evaluated the rheological properties across varying temperatures. Notably, an increase in graft percentage was observed, leading to a shift in the transition temperature from 34 °C to 37 °C [78].

#### 5.2.3. PNIPAM Crosslinking Through Monoacryloxyethyl Phosphate

Monoacryloxyethyl phosphate (MAEP) is a water-soluble phosphoric acid ester monomer that includes bis(2-acryloxyethyl) phosphate. Researchers developed chemically crosslinked thermoresponsive hydrogels of PNIPAM with MAEP and acrylamide, in which the LCST could elevate to physiological temperature. The formation of a thermogelling macromer (TGM) led to enhanced LCSTs above physiological temperatures. The phosphate group in MAEP plays a crucial role in promoting the postpolymerization attachment of hydrophobic, chemically crosslinkable, and transparent groups, allowing for the monitoring of the LCST reduction process below physiological temperatures [79].

## 6. Routes of Administration

### 6.1. Parenteral Route

**Figure 2:**
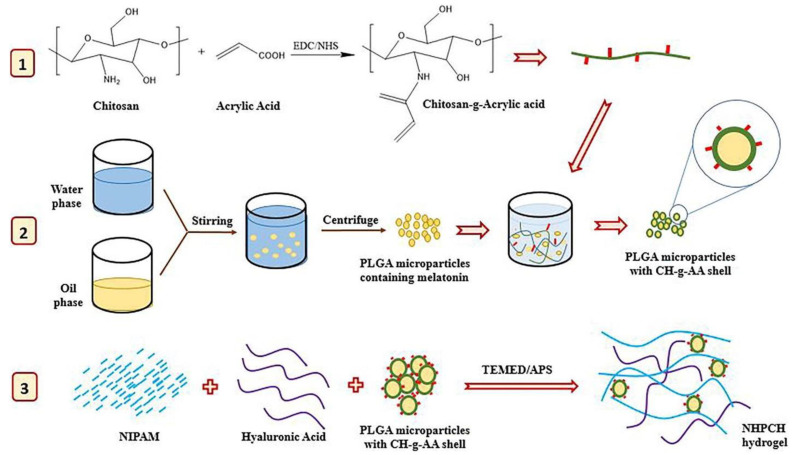
The whole procedure for hydrogel preparation: 1. Chitosan-g-acrylic acid is synthesized to generate C=C groups on the chitosan chains. 2. PLGA particles containing melatonin are synthesized using a microemulsion method; then, the surface of these particles is covered by CH-g-AA. 3. The polymerization of NIPAM in the presence of hyaluronic acid and particles as a crosslinker occurs to produce an injectable NHPCH hydrogel, Reprinted with permission from [83], copyright 2019, Elsevier.

Injectable hydrogels can be developed by various methods such as ionic crosslinking, photopolymerization, and changes in pH or temperature [80,81]. Injectable polymeric systems for drug delivery offer various advantages, including improved bioavailability, lower costs compared to surgical interventions, etc. Moreover, thermosensitive polymers can be used during network formation, overcoming challenges associated with post-loading methods [82]. Researchers developed an injectable thermoresponsive hydrogel made of PNIPAM and HA, incorporating CS-g-acrylic acid-coated PLGA (ACS-PLGA) micro/NPs at varying concentrations. These particles aimed to enhance cartilage regeneration by crosslinking PNIPAM for improved mechanical properties, mimicking natural cartilage, and using the PLGA core for the controlled release of the chondrogenic molecule melatonin (Figure 2). The selected hydrogel, which exhibited minimal syneresis and a high compression modulus, showed good integration with natural cartilage and an interconnected porous structure in SEM images. Cytotoxicity tests with mesenchymal stem cells demonstrated that the hydrogel supported cell growth better than tissue culture polystyrene. Histological analysis revealed that melatonin treatment enhanced glycosaminoglycan (GAG) production. With its improved mechanical properties, minimal syneresis, sustained drug release, and high bioactivity, this injectable hydrogel shows strong potential for cartilage tissue engineering [83].

Recently, Chen et al. developed a thermoreversible hydrogel by grafting PNIPAM-COOH onto chitosan, forming CS-g-PNIPAM (CPN), which was further modified with HA to produce HA-CPN. All three, PNIPAM-COOH, CPN, and HA-CPN, formed injectable, free-flowing solutions that underwent a reversible sol-to-gel transition above 30 °C when the polymer concentration exceeded 5%. The study analyzed the chemical and temperature-sensitive properties of hydrogels, including rheological behavior, phase transition, and water content. Adding CS increased the mechanical stiffness, while HA conjugation reduced it. Grafting CS and HA also improved water content and reduced volume contraction during phase transition. In vitro studies with chondrocytes and meniscus cells in the HA–CPN hydrogel showed improved cell morphology, proliferation, and differentiation, along with increased extracellular matrix content and mechanical properties, indicating active tissue formation [84].

To address drug resistance in cancer treatment, the thermoresponsive injectable gel effectively delivers medication to the targeted area. Therefore, Liu and teammates synthesized a thermoresponsive copolymer, alginate-g-PNIPAM (alginate-g-PNIPAM), by attaching PNIPAM to alginate. PNIPAM with varying molecular weights and narrow distribution was synthesized using ATRP. The copolymer forms self-assembled micelles in water or PBS above its critical micellization temperature and transforms into thermoresponsive hydrogels at body temperature (37 °C) when at a concentration of 7.4 wt%. The hydrogels, loaded with the anticancer drug doxorubicin (DOX), released DOX-loaded micelles in a controlled manner, improving drug uptake in multidrug-resistant cells and enhancing cancer cell killing. These injectable alginate-g-PNIPAM-HYDs show great potential for overcoming drug resistance in cancer treatment [85].

### 6.2. Transdermal Route

**Figure 3:**
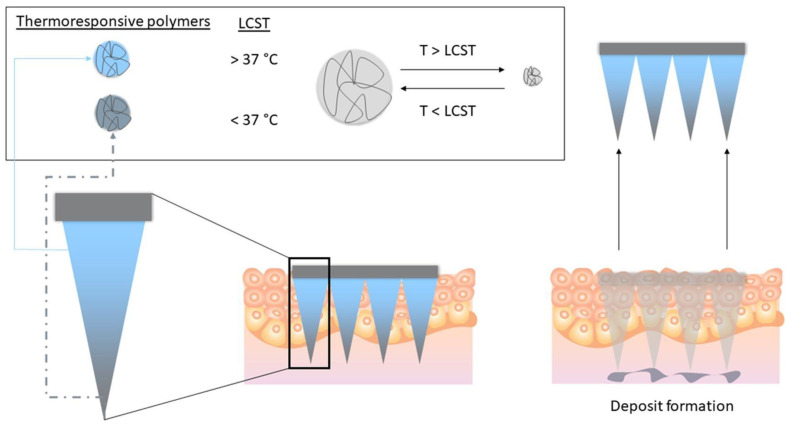
Thermoresponsive PNIPAM-based dissolving microneedles exhibit controlled drug deposition, with Bis-PNIPAM formulations forming a sustained dye deposit at ≈200 µm skin depth after 4 h, Reprinted from [94], licensed under CC BY 4.0.

Thermosensitive hydrogels play a key role in transdermal drug delivery systems (TDDSs) because of their outstanding biocompatibility, improved mechanical properties, and capability to offer sustained drug release [86,87]. Microneedles have gained significant attention as an effective method for use in TDDSs [88,89,90,91]. This study investigates PNIPAM as a novel material for a thermally responsive microneedle drug delivery system [92,93]. PNIPAM undergoes a reversible transformation from a swollen state at lower temperatures to a more hydrophobic state at higher temperatures, enabling controlled drug release. The findings show that dissolving microneedle patches made from PNIPAM, enhanced with BIS PNIPAM (a crosslinked variant), exhibit improved mechanical properties, with only about a 10% reduction in height. While microneedles made solely from PNIPAM can be developed, they lack adequate mechanical strength, requiring the incorporation of polymeric excipients like PVA. This study also found that the thermoresponsive polymer did not significantly affect the needles’ insertion properties, as all formulations penetrated ex vivo skin to a depth of 500 µm. Microneedles were loaded with a model dye, and their deposition was tracked using multiphoton microscopy, revealing a deposit at approximately 200 µm (Figure 3). Importantly, only the crosslinked PNIPAM formulations demonstrated significant dye accumulation in the skin after 4 h, regardless of the excipient used, while the non-crosslinked formulations did not show this effect. Overall, this study highlights the potential of PNIPAM-based dissolving microneedles for delivering NPs into the dermis for sustained drug release [94].

A research team developed a dual-responsive hydrogel for drug delivery, incorporating pH-sensitive microparticles that encapsulate betamethasone dipropionate (BD) and hexane extract from *Boesenbergia rotunda*. The PLLA-V6-OEG3 microparticles, made by emulsification–solvent evaporation, were spherical, monodisperse, and negatively charged, with 98% BD encapsulation efficiency. These microparticles released BD the most quickly at mildly acidic pH (6.0). The PNIPAM-BIS hydrogel, crosslinked by UV irradiation, showed significant improvements in the swelling ratio (>600%) and phase transition (>250%). Skin studies demonstrated effective BD delivery, with a skin deposition of 31.01 ± 1.78 μg/cm^2^ from the hydrogel. Additionally, *B. rotunda* extract, particularly its main component pinostrobin, enhanced BD release. Overall, the PNIPAM-BIS3 hydrogel loaded with PLLA-V6-OEG3 microparticles and *B. rotunda* extract shows great potential for treating atopic dermatitis [95].

Similarly, another team developed double-crosslinked IPN hydrogels made from temperature-sensitive PNIPAM and pH-sensitive HA using radical polymerization and Michael addition. These developed hydrogels were evaluated for various physicochemical characteristics. The double-network structure of the HA/PNIPAM-IPN hydrogel was confirmed using FT-IR and 13C NMR analysis. Swelling ratio measurements demonstrated pH and temperature sensitivity, which were influenced by the amount of crosslinking agent used. Texture analysis and rheometry revealed that the IPN hydrogel with 3% crosslinker content exhibited the best adhesion and a stable crosslinked network, making it suitable for luteolin loading. Skin permeation studies showed that HA-PNIPAM-IPN-HYD effectively delivered luteolin to various skin layers. Additionally, cytotoxicity tests indicated that the hydrogel was non-toxic for skin application. They concluded that these IPN hydrogels represent a promising TDDS for luteolin in treating psoriasis [62].

### 6.3. Ocular Route

**Figure 4:**
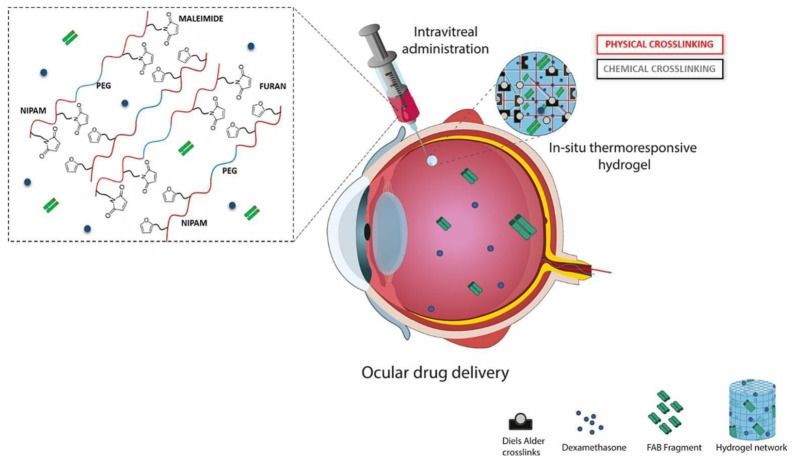
Thermoresponsive Diels–Alder stabilized hydrogels enable sustained ocular drug delivery of corticosteroid and anti-VEGF Fab fragment, providing controlled release for potential treatment of ocular diseases, Reprinted from [101], licensed under CC BY 4.0.

The ocular route encounters challenges in retaining drugs in the eye due to low corneal permeability, frequent blinking, and tear drainage [96,97,98]. This issue can be addressed by using hydrogels in liquid or gel form, which improves ocular drug delivery. Additionally, hydrogels enhance topical drug delivery by retaining significant amounts of water, helping to maintain a moist environment [99]. Their biocompatible nature makes hydrogels essential for controlled drug delivery systems (CDDSs) and applications in nanotechnology [100]. Ilochonwu et al. designed a novel thermosensitive hydrogel system for drug delivery in ocular therapy. Two types of ABA triblock copolymers were synthesized, incorporating furan and maleimide groups, named PNIPAM-co-HEA/Furan)-PEG6K-P(NIPAM-co-HEA/Furan) (PNF) and PNIPAM-co-HEA/Maleimide)-PEG6K-P(NIPAM-co-HEA/Maleimide) (PNM). The hydrogels were formed by mixing aqueous solutions of PNF and PNM, followed by incubation at 37 °C, resulting in an immediate sol–gel transition. This in situ hydrogel formation was further confirmed in ex vivo rabbit eyes after intravitreal injection. The hydrogel network was created through the physical self-assembly of PNIPAM blocks and catalyst-free furan–maleimide Diels–Alder (DA) chemical crosslinking in the polymer’s hydrophobic regions (Figure 4). Rheological analysis showed a sol–gel transition at 23 °C, with DA crosslinking occurring over 60 min as the temperature increased from 4 °C to 37 °C. At 37 °C, the hydrogels remained stable for at least one year in phosphate buffer at pH 7.4 but degraded at higher pH levels due to the hydrolysis of ester bonds in the crosslinks. The hydrogel was loaded with either an anti-VEGF antibody fragment (FAB) or the corticosteroid dexamethasone (DEX) by dissolving or dispersing them in the precursor solution. The FAB fragment released over 13 days, with initial bursts of 46%, 45%, and 28% for hydrogels containing 5%, 10%, and 20% weight, respectively, due to gel dehydration. In contrast, dexamethasone was nearly completely released over 35 days, likely because it was solubilized within the gel’s hydrophobic regions. The thermosensitive gels also demonstrated good cytocompatibility with RAW 264.7 and ARPE-19 cells. This research study concluded that the PNF-PNM thermogel could be an effective formulation for the sustained release of bioactive agents in the treatment of posterior segment eye disease [101].

Similarly, mixed thermoreversible gels were effectively designed by incorporating the thermosensitive polymer PNIPAM into fibrillar nanostructures using short peptides. When the temperature exceeds the LCST of PNIPAM, the polymer collapses into globular particles that act as crosslinks, connecting peptide nanofibrils and forming a hydrogel. Since this process relies on physical interactions, the hydrogels can reversibly transition between the sol and gel states with temperature changes. The antibacterial peptide G(IIKK)3I-NH2 was efficiently encapsulated by introducing its solution during the sol phase at lower temperatures, followed by increasing the temperature for gelation. This hydrogel enabled the sustained and controlled release of the peptide over time. By utilizing peptide nanofibrils as 3D scaffolds, these thermoresponsive hydrogels mimic the extracellular matrix and offer significant potential for injectable drug delivery and tissue engineering applications [102].

To sustain the delivery of proteins to the ocular region, Egbu et al. developed thermosensitive injectable gels. HA is a biodegradable material commonly used in the clinical area, while PNIPAM is a stimuli-responsive polymer with an LCST that can be achieved under physiological conditions. Two gel systems containing HA and the antibody infliximab (INF) were developed: (i) HA-Tyr, with 1% and 5% tyramine-substitute HA, enzymatically crosslinked in the presence of INF and (ii) PEGDA-PNIPAM-HA-INF, formed by chemically crosslinking NIPAAM, HA, and INF with 1% and 3% poly(ethylene glycol) diacrylate (PEGDA). The PEGDA-PNIPAM-HA-INF hydrogels exhibited high LCSTs (31.4 ± 0.2 to 35.7 ± 0.3 °C). INF-loaded gels with lower crosslinking densities (1% PEGDA-PNIPAM-HA and 1% HA-Tyr) had lower elastic and viscous moduli, leading to different swelling ratios compared to gels with higher crosslinking densities (3% PEGDA-PNIPAM-HA-INF and 5% HA-Tyr-INF). All gels showed the sustained release of INF in an in vitro human eye outflow model. The 1% PEGDA-PNIPAM-HA-INF hydrogel released the slowest, with 24.9 ± 0.4% of INF released by day 9, outperforming the free drug. This study suggests PEGDA-PNIPAM-HA has great potential for developing formulations aimed at prolonging intraocular protein release [103].

## 7. Biomedical Applications of PNIPAM-HYDs

### 7.1. Cancer Therapy

Despite significant advancements in cancer research, advanced cancer remains an incurable condition [104,105,106,107,108,109,110]. Ongoing studies aim to develop new formulations that improve drug efficacy while reducing side effects [111,112,113,114,115,116]. Hydrogel-based systems for localized, sustained drug release have been proposed to minimize off-target effects, with thermoresponsive hydrogels attracting particular interest [117,118]. These hydrogels transition from a liquid at 25 °C to a gel at body temperature (37 °C), making them promising for in situ sustained drug release in cancer treatment [119].

#### 7.1.1. Anticancer Agents’ Delivery

The PNIPAM polymer has an LCST of around 37 °C, and below the LCST, it is fully soluble in water, but above the LCST, it becomes less soluble or may even collapse. The LCST is close to the temperature at which many physiological processes occur. The literature reported that PNIPAM is considered a promising material for creating targeted drug delivery systems [120]. The sustained and targeted delivery of drugs is a key focus in drug delivery research. To address challenges in this field, Mohan et al. developed a stimuli-sensitive PNIPAM-based copolymer hydrogel for delivering various drugs. They synthesized a melamine-functionalized poly-N-isopropyl acrylamide-co-glycidyl methacrylate copolymer (PNIPAM-co-pGMA-Mela) and tested its dual pH and temperature-responsive drug release with ibuprofen (hydrophobic) and 5-fluorouracil (hydrophilic) under different conditions. The hydrogel released nearly 100% of both drugs at pH 4.0 and 45 °C. The in vitro test results showed that the hydrogel system effectively responded to both pH and temperature stimuli, releasing approximately 100% of Ibu and 5-Fu at pH 4.0 and 45 °C. Furthermore, an MTT assay and hemocompatibility analysis indicated that the PNIPAM-co-pGMA-Mela hydrogel is biocompatible and hemocompatible, suggesting its potential for drug delivery applications [121].

**Figure 5:**
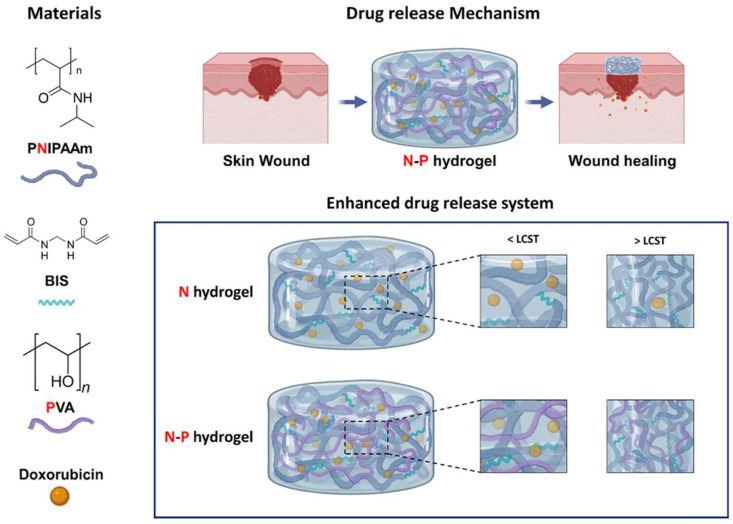
Schematic illustration of temperature-responsive N–P hydrogel, Reprinted from [122], licensed under CC BY 4.0.

A semi-IPN is composed of two or more networks that partially intertwine within a combined polymer structure without undergoing crosslinking, whereas a full IPN completely intertwines the networks. The authors estimated the effects of a semi-IPN hydrogel on cancer. Researchers fabricated the semi-IPN hydrogel to deliver DOX, which consists of a non-crosslinked network of NIPAM and poly(vinyl alcohol) (PVA) against melanoma cells. PVA was selected for its strong adhesive properties, biocompatibility, and non-toxicity, helping to mitigate the hydrophobicity often seen in PNIPAM polymers and enhancing the internal structure’s density. This modification enables better control over the release rates of substances from hydrogel. The NIPAM-PVA (N−P) (Figure 5) hydrogel exhibited temperature-responsive behavior, with an LCST of approximately 34 °C. The addition of PVA enhanced porosity and sped up drug release (78%). In vitro biocompatibility tests showed that the hydrogel is non-toxic and promotes cell proliferation. Moreover, the N−P hydrogel exhibited strong anticancer activity against melanoma cells, due to its rapid drug release. This N−P hydrogel system shows great promise for controlled drug delivery systems (CDDSs) and applications in skin regeneration and cancer therapy [122].

Similarly, Santhamoorthy et al. developed a curcumin (Cur) hydrogel using NIPAM and acrylamide (AAm) as comonomers. The thermoresponsive phase conversion behavior of the PNIPAM-co-PAAm hydrogel was examined, revealing that it reacts to temperature changes. The in vitro drug loading and release of Cur were studied under varying pH and temperature conditions. The PNIPAM-co-PAAm hydrogel showed pH and temperature-responsive drug release, with a loading efficiency of approximately 65% for Cur. Complete release occurred within 4 h at pH 5.5 and 40 °C. Cytotoxicity tests on HepG2 liver cancer cells showed the hydrogel’s good biocompatibility, supporting its potential as a drug delivery carrier. The MTT assay further confirmed its biocompatibility, suggesting its promise for solid tumor-targeted drug delivery [123]. Eskandari et al. studied the controlled release of DOX by developing a PNIPAM thermosensitive hydrogel. Temperature-responsive hollow hydrogel particles were created by etching silica cores with hydrofluoric acid. Particles with higher crosslinking density had larger hydrodynamic diameters (Dh) above the gelation temperature (GCT), while those with lower crosslinking density showed larger Dh below the GCT. Particle sizes ranged from 200 to 700 nm, with PDI values around 0.2, indicating a wide size distribution. Hollow particles with lower crosslinking density had higher drug loading capacity and the slowest release of DOX, with release decreasing as the temperature increased from 25 to 40 °C [124].

The literature showed that PNIPAM-HYD enhances the solubility and release of Cur. As a proof of concept, Ayar and team aimed to develop a drug delivery system using PNIPAM-HYD and an appropriate solvent to improve the solubility and localized release of Cur. PNIPAM-HYD was synthesized through radical polymerization, and its chemical, mechanical, and physical properties and biocompatibility were evaluated for its use as an implantable and rechargeable drug reservoir. Cur was incorporated into the hydrogel during swelling using two different solvents: methanol (organic solvent) and PEG200 (polymeric solvent). The results showed that PEG200 significantly enhanced Cur solubility compared to methanol. Furthermore, the drug release profile revealed that PEG200 enabled a higher cumulative release of Cur (33.163 ± 0.319 μg/mL) over one week compared to methanol (8.765 ± 0.544 μg/mL). The Cur-loaded hydrogels were non-cytotoxic, and the PNIPAM/PEG combination demonstrated antibacterial effects within 12 h. These findings suggest that the PNIPAM-HYD with PEG200 is a promising drug delivery system for the preservation and sustained release of Cur as a hydrophobic drug [125].

#### 7.1.2. Photothermal/Photodynamic Therapy (PTT/PDT)

Phototherapeutic agents used in light-activated therapies are safe for treating various malignant tumors [126]. The two primary types of phototherapies are PTT, which inflicts localized thermal damage on targeted areas, and PDT, which produces reactive oxygen species (ROS) to induce localized chemical damage [127,128]. However, conventional phototherapies face a significant challenge in clinical settings due to phototoxicity, mainly resulting from the uncontrolled distribution of these agents within the body. To reduce the side effects of phototherapy and enhance its effectiveness, significant research has been dedicated to developing hydrogel-based phototherapy for tumor treatment. Using hydrogels as drug carriers enables the sustained release of phototherapeutic agents directly to tumor sites, which helps minimize their adverse effects [129].

Microgel-based drug delivery systems have gained attention but face challenges like low drug loading, leakage, quick elimination from the bloodstream, and limited tumor effectiveness. To address these issues, Jo et al. developed a near-infrared (NIR)-responsive PNIPAM–Prussian blue microgel (PNIPAM@PB). The PNIPAM–Prussian blue (PB) microgel showed enhanced stability and a higher capacity for PB loading through chemical bonding. By incorporating the model drug DOX, a PNIPAM@PB@DOX microgel was created, achieving a 26% loading capacity and 51% encapsulation efficiency (EE). Under 808 nm NIR laser exposure, the photothermal PB in the microgel increased the temperature above its LCST, triggering shrinkage and releasing 36% of the drug. This NIR-triggered release improved both photothermal and chemotherapy effects in vitro and in vivo, particularly against A549 lung cancer cells, outperforming the PNIPAM@PB microgel. The combined PTT and chemotherapy effectively killed cancer cells [130]. Baipaywad et al. developed PNIPAM-based nanogel systems incorporating graphene oxide (GO) for controlled in vitro drug delivery, leveraging GO’s unique properties to restore conductivity through oxidation. The photothermal effects of the PNIPAM/GO and PNIPAM-AAM/GO nanogels were enhanced, with hydrodynamic diameters of 471 nm and 297 nm, respectively, at 25 °C. The PNIPAM/GO nanogel released 70% of DOX through photothermal effects triggered by near-infrared irradiation. A Cell Counting Kit-8 assay confirmed that the PNIPAM composite-based nanogels were biocompatible, showing no significant cytotoxicity [131].

**Figure 6:**
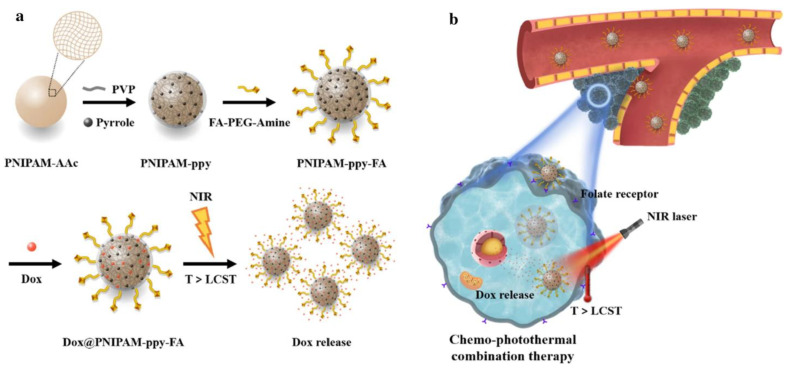
Schematic illustration of (**a**) synthesis of NIR light and thermo-triggered Dox@PNIPAM-ppy-FA nanocomposites and (**b**) application for enhanced chemo-photothermal combination therapy in breast cancer cells, Reprinted from [133], licensed under CC BY 4.0.

Howaili et al. developed a pH and thermal-responsive plasmonic nanogel (AuNP@Ng) for the delivery of Cur. AuNP@Ng was synthesized by grafting PNIPAM to CS using a chemical crosslinker to create a drug carrier system. The therapeutic efficacy of curcumin (Cur) was enhanced through photothermal therapy (PTT) using a plasmonic nanogel with a hydrodynamic size of approximately 167 nm. The nanogel provided sustained Cur release for up to 72 h, with dual thermo-pH-responsive release. Cytocompatibility tests on MDA-MB-231 breast cancer cells and non-tumorigenic MCF 10A cells confirmed its safety. The nanogel was more efficiently internalized by cancer cells, entering via an endosomal pathway, and showed dose- and time-dependent Cur delivery. The AuNP@Ng/Cur system improved chemotherapy effectiveness when exposed to NIR lasers (808 nm), making it promising for dual therapy combining drug delivery and PTT [132]. In another study, researchers developed targeted, multifunctional PNIPAM-based nanocomposites for chemo-photothermal therapy (chemo-PTT) against breast cancer. Acrylic acid (AAc) was added to PNIPAM to increase the transition temperature, shifting the LCST to 42 °C. Polypyrrole (ppy) NPs were incorporated to generate a photothermal effect under NIR laser (808 nm) exposure. Folic acid (FA) was conjugated to PNIPAM for targeted delivery to cancer cells. Drug release from PNIPAM-ppy-FA nanocomposites was triggered by NIR-induced temperature changes. The nanocomposite was internalized by MDA-MB-231 cells via folate-receptor-mediated endocytosis, enhancing cancer treatment through combined chemo-PTT effects (Figure 6) [133].

Recently, Algi and team fabricated a hydrogel using PNIPAM and squaraine dye (Sq1) as the polymer and the crosslinker, respectively. The authors evaluated the fluorescein release behavior of the Sq1@PNIPAAm hydrogel. Furthermore, Sq1@PNIPAAm hydrogels can be used as photosensitizers pertinent to photodynamic therapy (PDT). The results showed that the hydrogel possesses favorable biological safety for use in in vitro anticancer studies. In vitro experiments confirmed that Sq1@PNIPAAm hydrogels could kill over 40% of cancer cells. The results revealed that Sq1@PNIPAAm enabled photodynamic therapy. Moreover, fluorescein loading into Sq1@PNIPAAm was possible, and it could be used to accomplish temperature-controlled on-demand release. Given the abundance of low-cost, commercially accessible monomers available for use in hydrogel synthesis, this method offers access to a wide range of functional hydrogels for use in biomedical applications [134].

### 7.2. Wound Healing

Wound healing typically involves four interconnected phases: hemostasis, inflammation, proliferation, and remodeling [135]. Establishing a clean, moist environment for the wound can speed up healing while minimizing inflammation. Hence, hydrogel dressings can enhance the healing process by providing a moist environment that encourages tissue regeneration [136]. By implementing this idea, Jing and team developed PNIPAM-HYD crosslinked with composite NPs to improve wound healing. Researchers developed a novel type of thermosensitive hydrogel to enhance the scavenging property of reactive oxygen species (ROS) and accelerate wound healing. Nano-CeO2 was evenly distributed on the surface of mesoporous silica (MSN), and the resulting nanocomposite particles were physically crosslinked with PNIPAM to create a thermoresponsive hydrogel known as MSN-CeO2@PNIPAM (PMCTH). PMCTH was thoroughly evaluated for various characteristics. The findings showed that the hydrogel-based system remained stable over time with low biological toxicity and a phase transition temperature near normal body temperature. When applied to the skin, the PMCTH allowed MSN-CeO2 NPs to disperse within the hydrogel, reducing ROS-related damage and promoting blood vessel formation and tissue regeneration, thereby accelerating wound healing. Animal studies showed that a 40% mass ratio of CeO_2_ to MSN achieved a wound healing rate of 78% by day 10, significantly surpassing other experimental groups. This research provides a novel approach and experimental foundation for using functional hydrogels in wound healing [137].

In another study, novel hydrogel dressings with adjustable contractility were developed using N-isopropyl acrylamide, sodium alginate, and GO (P/SA/GO). The CS solution was used as a bridging polymer to enhance tissue adhesion to PNIPAM-HYD, which exhibited self-shrinking properties controlled through NIR thermal stimulation. Adding CS improved the adhesion strength between the tissue and dressing (7.86 ± 1.22 kPa) with adjustable localized adhesion. Mouse skin defect tests showed that temperature adjustments enabled suture-free wound closure in early healing stages, while the dressings promoted scar-free healing by reducing inflammation and collagen buildup. These double-crosslinked PNIPAM-HYD dressings with adjustable adhesion and contractility offer a promising approach for effective wound healing [138]. Liu et al. developed cotton bandages infused with a PNIPAM/GO/nano silver hydrogel (PNIPAM/GO-Ag/COT) and examined their healing effects on deep second-degree burn wounds in rats, along with changes in inflammatory factors. The results showed that the PNIPAM/GO-Ag/COT dressing reduced TNF-α expression by about 18% and increased bFGF levels in the wound tissue compared to the blank group (without any dressing). The dressing also improved the wound healing rate by 30%, reaching 58% by day 14, compared to the gauze-treated control group. The study concluded that the PNIPAM/GO-Ag/COT dressing promotes burn wound healing by reducing inflammation, encouraging collagen deposition, and enhancing bFGF expression [139].

**Figure 7:**
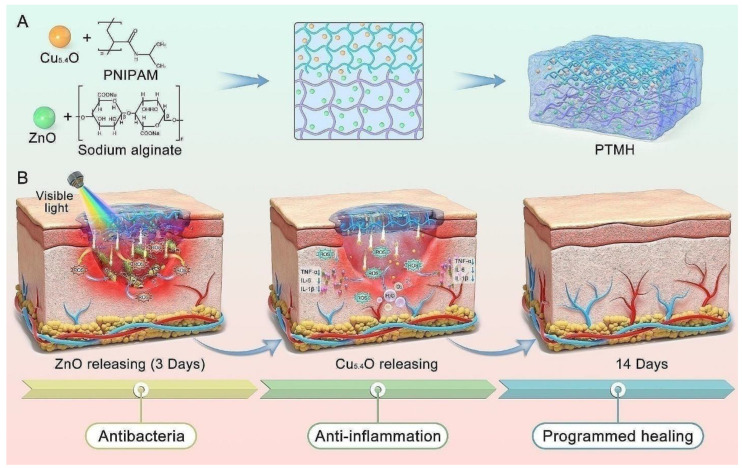
A schematic of the design, structure, and therapeutic process of PTMH for impaired wound healing. (**A**) A dual-layer hydrogel with sodium alginate (SA)-loaded zinc oxide (ZnO) nanoparticles and poly(N-isopropylacrylamide) (PNIPAM)-loaded Cu5.4O ultrasmall nanozymes was designed. (**B**) The programmed regulation process in the mouse model of diabetic wounds with P. aeruginosa infection, including anti-infection with ZnO via the generation of reactive oxygen species (ROS) and anti-inflammation, as well as angiogenesis promotion by Cu5.4O ultrasmall nanozymes. PTMH, programmed time-released multifunctional hydrogel, Reprinted from [140], licensed under CC BY 4.0.

Peng and teammates developed a time-released multifunctional hydrogel (PTMH) designed to dynamically regulate the wound inflammatory microenvironment at different healing stages. The dual-layer hydrogel combined sodium alginate (SA) with zinc oxide (ZnO) NPs and PNIPAM loaded with Cu5.4O ultrasmall nanozymes. The PTMH addressed early-stage bacterial infections by generating ROS from ZnO under visible light while gradually degrading its lower layer. When the upper layer contacts the wound tissue, it releases Cu5.4O nanozymes to neutralize excess ROS, reduce inflammation, and promote the transition from the inflammatory to the proliferative phase. Additionally, the Cu5.4O nanozymes improved angiogenesis and enhanced oxygen and nutrient delivery to the damaged tissue (Figure 7). They showed that PTMHs notably sped up the healing of diabetic wounds with bacterial infections in mice, displaying strong antibacterial and anti-inflammatory effects over time [140].

Chronic wounds in diabetic patients remain one of the most challenging issues in clinical skin injury repair. The microenvironment of a wound directly influences the healing process, but traditional dressings have limited effectiveness in managing this environment and promoting healing. To tackle this problem, Li and team developed a thermosensitive drug-controlled hydrogel that self-adjusts the wound microenvironment. This hydrogel is made from sodium alginate (SA), Antheraea pernyi silk gland protein (ASGP), and PNIPAM, enabling intelligent drug release to support skin regeneration. PNIPAM exhibits favorable thermal transition temperatures ranging from 31 °C to 34 °C, causing the hydrogel to contract and expel drugs and water molecules when the LCST is exceeded, which imparts smart drug release capabilities. The maximum adhesion strengths recorded were 31.48 ± 2.01 kPa for glass, 39.87 ± 1.95 kPa for polycarbonates, 25.06 ± 2.23 kPa for stainless steel, and 38.56 ± 2.16 kPa for plank and rubber. The inclusion of ASGP enhances biocompatibility and provides hydrogels with adhesive properties. In vitro studies also demonstrate that these thermosensitive drug-controlled hydrogels exhibit excellent biocompatibility, supporting the adhesion and growth of human skin fibroblast cells. This research introduces a new strategy for smart drug-controlled hydrogels that react to temperature variations, improving wound healing by autonomously regulating the wound microenvironment [141].

### 7.3. Tissue Engineering

A key focus in advanced materials science is the creation of smart polymers that can autonomously change their physical or chemical properties in response to external stimuli [142,143]. PNIPAM-based smart hydrogels exhibit unique thermosensitive behavior near their LCST, making them highly suitable for biomedical applications like drug delivery, tissue engineering, and wound healing [34]. Various techniques have been developed to create 3D biomimetic scaffolds from hydrogels, which have recently gained popularity in tissue engineering. Khiabani and team evaluated a hydrogel made up of PCL blocks and PNIPAM-hydroxyethylmethacrylate (NIPAM-HEMA) acrylate blocks for the simultaneous delivery of two osteogenic growth factors, VEGF and BMP2. The physical properties of the PCL-P(HEMA-NIPAM) hydrogel were characterized. The initial release of the growth factors was rapid within the first 24 h, with 45% and 50% of each being released. By the end of the study period, 80% of BMP2 and 85% of VEGF had been released from the hydrogel scaffold. Cell viability measurements indicated rates of 91%, 205%, and 470% after 24 h and on the third and fifth days, respectively. SEM images showed that human dental pulp stem cells (hDPSCs) fully adhered to, colonized, and proliferated on the hybrid copolymer, demonstrating its non-toxic nature. The hydrogel exhibited no significant cytotoxicity toward hDPSCs, and the novel PCL-P-based scaffold, loaded with growth factors, serves as an effective matrix for hDPSCs and cell seeding, offering great potential for use in 3D constructs for bone tissue engineering and regenerative medicine [144].

In a similar approach, a new series of thermoresponsive biodegradable hydrogels (TBHs) was developed by combining N-isopropyl acrylamide (NIPAM) with two biodegradable crosslinkers: poly(ε-caprolactone) dimethacrylate (PCLDMA) and bisacryloylcystamine (BACy). The results showed that the properties were significantly influenced by temperature and the molar ratio of PCLDMA to BACy. Levofloxacin-loaded hydrogels (LVF-TBHs) were also created to investigate their stimuli-responsive release. The %CDR profile of LVF-TBHs showed slow, sustained release triggered by temperature and rapid release induced by reduction. At physiological pH and 37 °C, the TBHs biodegraded slowly in the presence of glutathione (GSH). With their uniform pore size, interconnected structure, degradable chemistry, and thermoresponsive properties, these TBHs hold great potential for tissue engineering scaffolds [145]. The electrochemical polymerization (ECP) of acrylic monomers has gained attention for creating biocompatible coatings for medical device electrodes. This study explored ECP as a method for fabricating highly porous PNIPAM–hydroxyapatite (HAp) composites for bone tissue regeneration. FT-IR analysis confirmed the successful conversion of NIPAM to PNIPAM through ECP. The PNIPAM-HAp scaffolds showed no cytotoxicity to MG63 cells, proving ECP’s viability for scaffold production. Additionally, the scaffolds were loaded with oxacillin to combat osteomyelitis, showing effective drug release and antibacterial activity against *S. aureus* and *P. aeruginosa*. ECP PNIPAM exhibited significantly higher drug loading (75.6 ± 0.62% *w*/*w*) and incorporation efficiency (86.7 ± 2.19% *w*/*w*), making ECP a promising method for developing non-toxic, biocompatible scaffolds for tissue engineering [146].

In another study, thermosensitive PNIPAM was attached to gelatin through ATRP. The PNIPAM-grafted gelatin (Gel–PNIPAM) was characterized using XPS, ATR-IR, and 1H NMR analyses. The Gel–PNIPAM solution underwent sol-to-gel conversion at physiological temperature and was tested as an injectable hydrogel for bone defect regeneration in a cranial model. It showed good biocompatibility and enhanced bone regeneration compared to the control group. The addition of rat bone mesenchymal stem cells (BMSCs) further improved regeneration. Micro-CT, histology, and immunohistochemistry demonstrated woven bone formation for 12 weeks in the hydrogel–BMSC group. The study concluded that Gel–PNIPAM is a promising injectable system for BMSC-based bone defect repair [147]. Mellati et al. developed a controllable and defined 3D microenvironment for the chondrogenic differentiation of mesenchymal stem cells (MSCs). In this study, PNIPAM polymers with degrees of polymerization of 100 and 400 (NI100 and NI400) were combined with CS porous scaffolds to create hybrids (CSNI100 and CSNI400). SEM images showed that the PNIPAM gel partially filled the CS pores while preserving the scaffold’s interconnected structure. MSCs were incorporated into the hybrids, and their proliferation and chondrogenic differentiation were evaluated. After seven days, cell viability in CSNI100 and CSNI400 were 54% and 108% higher than in CS alone. After 28 days, glycosaminoglycan and collagen content increased significantly in cell-laden CSNI400. These results indicate that the CS-PNIPAM hybrid, especially CSNI400, is effective for 3D stem cell culture and cartilage tissue engineering [148].

### 7.4. Ocular Diseases

Three-dimensional polymer networks, referred to as hydrogels, have a remarkable ability to absorb water and biological fluids [149,150]. The polymer matrix of hydrogel features contributes to this water-absorbing capability. The degree of polymer hydration is affected by both the polymer content and the type of aqueous medium used [151]. Hydrogels can take up a significant amount of water due to crucial crosslinks in their structure; however, instead of dissolving in the surrounding liquid, they swell [152]. The sustained delivery of DEX to the ocular area was evaluated by Blessing and team. A self-healing hydrogel was developed using a thermosensitive ABA triblock copolymer. In this study, dexamethasone was covalently bonded to a polymer via the copolymerization of methacrylate dexamethasone with NIPAM and N-acryloxysuccinimide using RAFT polymerization, with PEG functionalized with a chain transfer agent. The thermosensitive hydrogel was formed by mixing the polymer solution with cystamine at 37 °C, resulting in covalent crosslinking. The hydrogel released DEX via ester hydrolysis following first-order kinetics over 430 days at 37 °C, maintaining therapeutic levels in the vitreous for over 500 days. LC-MS analysis confirmed the release of dexamethasone in its native form. Cytocompatibility tests showed no toxicity to retinal cells at relevant concentrations. The injectability, long-lasting release, and cytocompatibility of the hydrogel make it a promising candidate for treating ocular inflammatory diseases [153].

Jones and teammates studied the role of excess PNIPAM in the aggregation of PNIPAM-coated AuNPs. They found that contrary to common belief, aggregation depends on the size and concentration of unbound “free” PNIPAM in the solution, which also increases solution turbidity, a common indicator of NP aggregation. The size of the PNIPAM used for coating and the concentration of the resulting polymer–AuNP composites had little effect on aggregation. In the absence of free PNIPAM, the polymer contracts in response to temperature increases, rather than causing NP aggregation, leading to no observable changes in turbidity or color [154]. Turturro et al. studied the effects of crosslinked thermoresponsive PNIPAM-HYD on retinal function, focusing on the safety of hydrogel injections for retinas. One week post-injection, they observed slight changes, including a 4% reduction in arterial and venous diameters, an 8% increase in venous blood velocity, and a 6% decrease in retinal thickness. Electroretinography showed a 15% reduction in a- and b-wave amplitudes, but all measurements returned to baseline within a week. The results suggest that PNIPAM-HYD injections have a minor, temporary effect on retinal function, supporting its potential for ocular drug delivery systems [155]. Lima and teammates evaluated the safety of intravitreal PNIPAM tissue adhesive injections in rabbits. Twelve rabbits received a 0.1 mL injection of 50% PNIPAM in one eye. Follow-up assessments, including color fundus photography, FA, OCT, and ERG, revealed mild to moderate inflammation in the first month, which resolved by the third month. The lens and cornea remained clear, with no retinal damage or neovascularization observed in OCT or FA. Histological analysis and ERG tests confirmed no retinal toxicity, indicating the safety of PNIPAM for intravitreal use. They concluded that the intravitreal injections of PNIPAM were non-toxic, suggesting that PNIPAM may be safe for use as a bioadhesive in certain retinal conditions [156].

### 7.5. Skin Diseases

**Figure 8:**
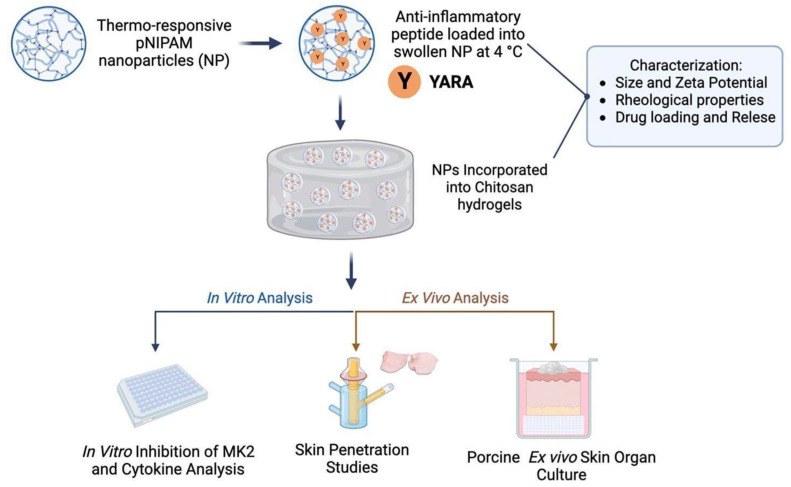
Thermoresponsive PNIPAM nanoparticles in chitosan hydrogels enhance YARA peptide delivery, sustaining release for up to 120 h and significantly reducing inflammatory cytokine levels in vitro and ex vivo, Reprinted with permission from [162], copyright 2023, Elsevier.

Thermoresponsive materials react to temperature changes, leading to physical and chemical changes in the material [157]. In drug delivery applications, polymers like PNIPAM and poloxamers (block copolymers of poly(ethylene oxide) and poly(propylene oxide)) demonstrate significant temperature sensitivity, releasing their drug contents when exposed to higher temperatures [158,159]. The LCST at which these changes occur depends on the concentration and chemical structure of the polymer, allowing for optimization through engineering. These phase transitions can be activated by the natural temperature gradient of the skin, as the temperature at the skin’s surface is typically lower than that of the body’s core [160]. Yurdasiper et al. aimed to develop triclosan nanogels made from PNIPAM with thermoresponsive properties, created through surfactant-free emulsion polymerization using N,N′methylene bisacrylamide as a crosslinker. Various characteristics were assessed, including antibacterial effectiveness against *Cutibacterium acnes*. The study found that the swelling capacity of nanogel formulations increased with the addition of triclosan and ethanol. At 25 °C, all formulations exhibited viscoelastic behavior, with a higher elastic modulus (G′) compared to 37 °C. G′ values also increased with higher concentrations of hydrophilic monomers. The encapsulation efficiency (EE) was 47.4% to 63.7%. The nanogel formulation showed superior antibacterial effectiveness against *C. acnes* compared to a commercially available control. The results indicate that PNIPAM-based triclosan nanogels have an appropriate size and rheological properties for dermal use. Their potent antibacterial activity makes this thermoresponsive nanogel formulation a promising alternative to conventional treatments for acne vulgaris [161]. Similarly, another team focused on loading a cell-penetrating MK2 inhibitor peptide into hollow thermoresponsive NPs (PNIPAM), which were then incorporated into CS-HYD (Figure 8). The NPs exhibited a high loading efficiency of over 50%, and SEM confirmed that the hydrogels remained porous after NP incorporation. Both NPs and hydrogels provided sustained YARA release for up to 120 h. They delivered significantly more YARA into the viable layers of porcine skin in vitro compared to the non-encapsulated compound, under both intact and impaired barrier conditions. Additionally, YARA-loaded NPs and H-NP-YARA reduced inflammatory cytokine levels in human keratinocytes by up to 20 times, and in an ex vivo skin model, cytokine levels decreased by up to 17 times with H-NP-YARA compared to the drug in solution. These results suggest that CS hydrogels with YARA-loaded NPs offer a promising formulation for treating atopic dermatitis [162]. Another research team explored a self-developed method involving a two-step photoinduced polymerization to create crosslinked polyacrylamide (CLPAM) soft hydrogel NPs, measuring 5–10 nm in diameter, which were grafted with PNIPAM chains. The ultrafine, water-swollen CLPAM@PNIPAM core–shell NPs, with diameters ranging from 20 to 35 nm, displayed temperature-sensitive behavior. Upon heating, the NPs quickly aggregated at around 33–34 °C, forming clusters about 120 nm in size. As the temperature increased to 45 °C, the size reduced to approximately 100 nm due to the formation of hydrophobic shell layers and the shrinkage of PNIPAM chains. This shrinkage caused the soft PAM cores to expel water and contract. During cooling, the contracted cores trapped within the aggregates led to early dissociation. These hydrophilic hairy CLPAM@PNIPAM particles show potential for targeted drug delivery systems (CDDSs) and other smart biomaterial applications [163]. In addition to pigment absorption and reflection by periodic photonic structures, many natural species achieve whiteness through light scattering. This study presents a PNIPAM-HYD network with interconnected channels (ch-PNIPAM) that displays temperature-induced bright whiteness. By removing agarose from a semi-IPN of agarose and PNIPAM, the hydrogel remains transparent at room temperature but turns white above 35 °C. Compared to conventional PNIPAM, ch-PNIPAM shows an 80% increase in reflectance and a phase transition speed 18 times faster. Nanoscopic channels facilitate water diffusion during phase transitions, forming smaller pores and enhancing whiteness. This rapid, photothermally triggered response makes ch-PNIPAM a promising material for skin applications [164].

### 7.6. Other Diseases

A major challenge in RNA interference (RNAi)-based therapies is delivering effective doses of nucleic acids, like siRNA, to targeted tissues while minimizing off-target effects [165,166]. To tackle this issue, hydrogels can serve as matrices for the localized and sustained release of siRNA. Fliervoet and team created polyplexes using siRNA and poly(2-dimethylaminoethyl methacrylate) (PDMAEMA)-based polymers, which were incorporated into a thermosensitive hydrogel to enhance the local release of siRNA. A multifunctional NPD triblock copolymer, consisting of thermosensitive PNIPAM (N), hydrophilic PEG (P), and cationic PDMAEMA (D), was used to assess its binding properties with siRNA, with a non-thermosensitive PD polymer as a control. Both polymers formed small polyplexes (10–20 nm) at an N/P charge ratio of 5 or higher. When siRNA was loaded into the thermosensitive PNIPAM–PEG–PNIPAM-HYD, it released more gradually and sustainably compared to free siRNA, with polyplexes being released over 128 h. The polyplexes remained stable and biologically active, effectively transfecting FaDu cells in vitro. This study demonstrates the potential of polyplex-loaded thermosensitive hydrogels for localized, sustained siRNA release, offering promising applications in tumor treatment and gene therapy [167].

**Figure 9:**
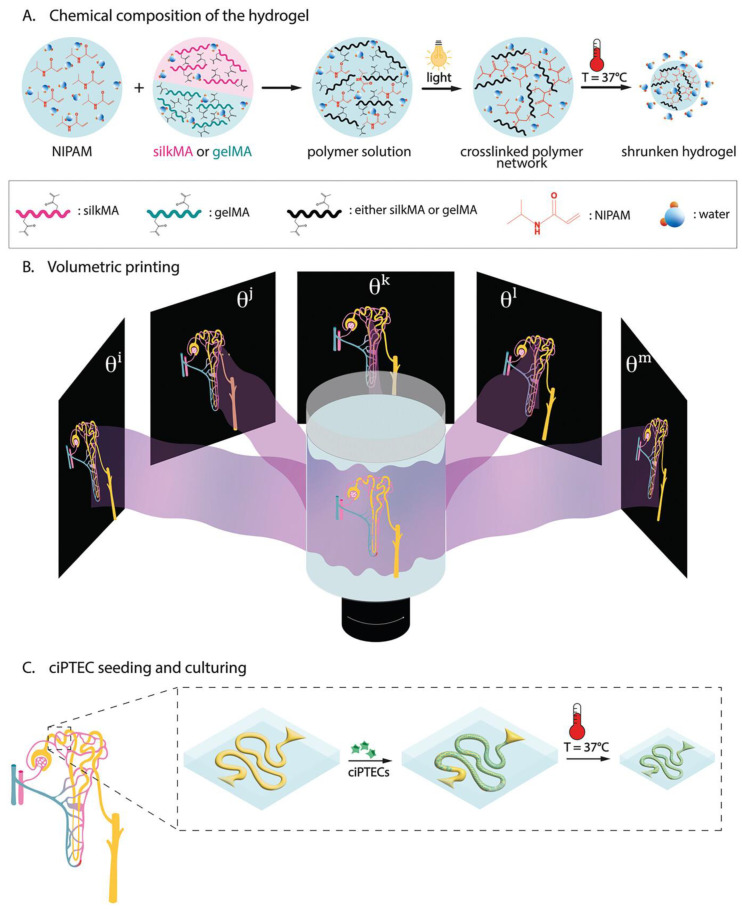
Schematic representation of overall process. (**A**) Chemical composition of 2 hydrogels gelMA-pNIPAM and silkMA-pNIPAM. (**B**) Overview of tomographic volumetric printing process. (**C**) Cell seeding using human conditionally immortalized proximal tubule epithelial cells (ciPTECs) inside hollow tubules printed with tomographic volumetric printer in gelMA-pNIPAM or silkMA-pNIPAM hydrogels, Reprinted from [169], licensed under CC BY 4.0.

In one study, researchers explored the potential of a thermosensitive PNIPAM-HYD for treating Parkinson’s disease (PD), which is often associated with mitochondrial dysfunction, including increased ROS production and impaired energy metabolism. Overcoming the challenge of the blood–brain barrier (BBB) is crucial for effective intracerebral drug delivery. To address this, a drug delivery platform, MAG-NCs@Gel, was developed by incorporating magnolol (MAG) nanocrystals (MAG-NCs) into a non-invasive thermosensitive PNIPAM-HYD, aimed at enhancing the delivery of therapeutic agents to the brain. The MAG-NCs@Gel system significantly enhanced drug solubility, nasal cavity retention, and brain targeting efficiency. Continuous intranasal delivery allowed MAG to cross the blood–brain barrier (BBB) and reach dopaminergic neurons, effectively reducing the symptoms of MPTP-induced Parkinson’s disease. The hydrogel’s LCST behavior improved its viscoelasticity, aiding MAG-NCs’ BBB penetration. This system normalized ROS and ATP levels in dopaminergic neuron mitochondria, reversed mitochondrial dysfunction, and improved behavior in PD mice, all without harming healthy tissues [168]. This study investigates the use of PNIPAM’s temperature-responsive properties to create complex, high-resolution tubular structures for tissue engineering. The approach involves a shrinking mechanism and combines two thermosensitive hydrogels—gelatin methacryloyl (gelMA) and silk fibroin methacryloyl (silkMA)—with PNIPAM (Figure 9). This study found that the temperature-controlled shrinking of PNIPAM-HYDs, combined with gelMA and silkMA, could be precisely adjusted between 33 and 37 °C. The size reduction enhanced mechanical properties, increasing the compressive modulus by 2.8-fold for gelMA-PNIPAM and 5.1-fold for silkMA-PNIPAM at 37 °C. Volumetric printing improved resolution, achieving 20% enhancement for positive features and 70% for negative features. GelMA-PNIPAM showed better cell compatibility, improving adhesion and viability. These thermosensitive hydrogels demonstrated potential for creating high-resolution, intricate tubular structures, offering a promising approach for in vitro tissue model development [169].

### 7.7. Diagnostic Applications

**Figure 10:**
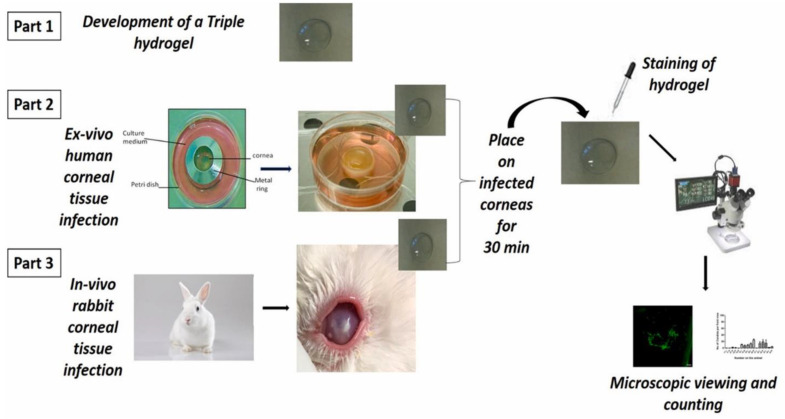
A schematic diagram of the overall strategy used for the evaluation of ligand-modified PNIPAM attached to a hydrogel for developing a device for the rapid detection of bacteria and fungi from corneal ulcer cases, Reprinted from [174], licensed under CC BY 4.0.

Research on photothermal agents has been a key focus in therapy and sensing, with various types synthesized, including noble metal, carbon-based, metallic compounds and organic nanomaterials [170]. Noble metal photothermal nanomaterials are favored for photothermal sensing due to their customizable morphology, easy synthesis, and surface modification. Their ability to adjust local surface plasmon resonance (LSPR) enhances photothermal conversion efficiency, making them attractive for diverse applications. Compared to traditional sensing methods like fluorescence and chromatography, photothermal-based systems provide notable advantages [171]. Based on this concept, Zhang and team presented a multifunctional sensing platform with excellent photothermal capabilities integrated into a paper-based electro-chemiluminescent (ECL) biosensor for accurate interleukin-6 detection. This study developed a multifunctional sensing platform using MBene@TaSe2 composites, which trigger multi-signals upon laser exposure. The platform utilizes a thermoresponsive PNIPAM-HYD that releases malachite green dye for visual readout, enabling quick and precise analysis from a single drop of sample. Employing a one-step sandwich immunoreaction simplifies the detection process. The platform was evaluated for clinical diagnosis by measuring IL-6 levels in human serum samples, with results comparable to those obtained using traditional ELISA (95.7% to 109.2% recovery rates). The findings highlight the platform’s high accuracy and potential for home monitoring and smart diagnostics [172]. Jiang and team developed an innovative polymer-based wound dressing designed to monitor the healing process. This dressing features a conductive, soft, temperature-responsive, antibacterial, and biocompatible hydrogel made from polyacrylic acid (PAA)-grafted PNIPAM, vinyl-based PAA, and silver nanowires (AgNWs). In this formulation, PAA-grafted PNIPAM acts as a temperature-responsive matrix, while PAM strengthens mechanical properties by forming a semi-IPN. The addition of AgNWs creates a conductive hydrogel network with antibacterial and sensing functions. Both hydrogels, with and without AgNWs, displayed a stable elastic network. Hydrogels with higher AgNW concentrations (1 or 2 mg/mL) showed strong bactericidal effects. Additionally, the hydrogel was connected to a Bluetooth module for the wireless monitoring of temperature changes, allowing for real-time infection detection. This study demonstrates a promising approach for improving wound care and medical diagnostics [173]. Shivshetty and team created a diagnostic device for corneal infections that can detect Gram-positive and Gram-negative bacteria, as well as fungi, without requiring a microbiology lab. They used branched PNIPAM modified with Vancomycin, Polymyxin B, or Amphotericin B to specifically target and bind to Gram-positive bacteria, Gram-negative bacteria, and fungi, respectively, all within a single hydrogel (Figure 10). This study presents a hydrogel system designed to specifically bind to various microorganisms, with a detection sensitivity of 10^4^ organisms per high-power field (100×). In ex vivo and animal cornea infection models, the hydrogel effectively captured bacteria, fungi, or a combination of both within just 30 min. The presence of the microbes was confirmed using conventional microbiological methods alongside fluorescently labeled ligands or dyes. This user-friendly hydrogel can be applied to infected eyes, allowing for the quick identification of different pathogens. Ultimately, this diagnostic tool aims to remove the need for specialized microbiologists, making it ideal for point-of-care applications [174]. The detection sensitivity of an electrochemical immunosensor is mainly influenced by the proximity to the sensing interface, and controlling this region is key to enhancing signal amplification. Similarly, a research team developed a photothermal-regulated sensing interface using an NIR-responsive hydrogel probe for the ultrasensitive detection of human epididymis protein 4 (HE4). The probe featured silver NP-deposited graphene oxide hybrids (AgNPs@GO), which served as electrochemical signal markers and photothermal transducers, encapsulated in PNIPAM-HYD. When exposed to NIR light, the AgNPs@GO hybrids converted the light to heat, causing the hydrogel to shrink and amplify the electrochemical signal. The shrinkage behavior improved the electrochemical reaction efficiency by regulating the interface, resulting in enhanced sensitivity compared to conventional sandwich immunoassays. The photothermal-induced interface regulation not only preserved a wider outer Helmholtz plane (OHP) region but also reduced it, further boosting sensitivity. Furthermore, the dual-mode signals provided high accuracy in detecting tumor markers. Overall, this detection approach demonstrates the potential of leveraging the photothermal effect in clinical diagnostics [175]. Some recent research findings for PNIPAM-based hydrogels for biomedical applications are depicted in Table 1.

## 8. Patents

Extensive research has emphasized the remarkable versatility of PNIPAM-based smart hydrogels across diverse biomedical applications. While a substantial number of patents have been filed to protect intellectual property in this innovative area, this discussion highlights a select group of notable patents. These examples, presented in Table 2, demonstrate the groundbreaking use of PNIPAM-HYDs in key biomedical fields such as drug delivery, advanced tissue engineering, and innovative wound dressing solutions, showcasing the translation of these technologies from experimental research to practical applications.

## 9. Conclusions and Future Perspectives

PNIPAM-HYDs have emerged as advanced stimuli-responsive materials due to their exceptional adaptability and multifunctional properties. They exhibit remarkable characteristics, including reversible transformations in volume, optical transparency, and surface hydrophilicity, making them ideal candidates for innovative biomedical applications. These hydrogels combine the temperature-responsive gelation properties of PNIPAM with the integration of diverse monomers, resulting in materials with acceptable biocompatibility and decent injectability.

A significant advantage of PNIPAM lies in its LCST, approximately 32 °C, which is remarkably close to the temperature of the human body. This proximity enables it to undergo phase transitions seamlessly under physiological conditions. This distinctive trait has made PNIPAM-HYDs highly versatile and valuable for various biomedical applications, including controlled drug delivery, advanced tissue engineering techniques, and innovative wound healing strategies. Although PNIPAM-HYDs hold great potential, their application is limited by challenges such as poor biodegradability and mechanical strength. To address these issues, researchers have made substantial progress through various strategies, including reinforcing mechanical properties, enhancing biodegradability, and designing copolymeric hydrogels by integrating PNIPAM with polymer blocks that exhibit distinct characteristics.

Future research is expected to focus on enhancing the mechanical strength, biodegradability, and responsiveness of PNIPAM-HYDs for biomedical applications. Innovations in multiresponsive hybrid hydrogels will enable more precise control over drug release, tissue engineering scaffolds, and wound healing materials. Advanced polymerization techniques, including controlled radical polymerization, ATRP, electron-beam irradiation, and RAFT polymerization, have been utilized to produce customized PNIPAM-modified surfaces. Additionally, bioconjugation strategies and nanocomposite integration could improve biocompatibility and structural stability. Combining PNIPAM with other materials has significantly broadened the functional scope of these hydrogels, facilitating multi-stimulus responsiveness and improved drug-loading capacities. Advances in 3D and 4D bioprinting may further facilitate the development of customized hydrogel-based implants and regenerative medicine solutions.

Despite these advancements, the clinical translation of PNIPAM-HYDs remains limited due to insufficient in vivo studies, long-term bioaccumulation concerns, and inadequate preclinical evaluation. To overcome these barriers, comprehensive toxicological investigations are imperative, focusing on physiological stability, potential toxicity, immune response, pharmacokinetics, biodegradability, and elimination pathways. The successful integration of PNIPAM-HYDs into clinical settings will require interdisciplinary collaboration, material innovation, and sophisticated fabrication techniques. With continued advancements, these hydrogels are poised to revolutionize biomedical technologies, paving the way for more efficient and targeted therapeutic solutions.

## Figures and Tables

**Table 1:** Recent investigations on PNIPAM hydrogels for various biomedical applications.

Composition of Hydrogel	Method of Preparation	Therapeutic Agent/Model Drug	Application	References
NIPAM, N,N′-methylene bis acrylamide, Poly-L-lactic acid	UV irradiation	Betamethasone dipropionate, hexane extract derived from *Boesenbergia rotunda*	Atopic dermatitis	[95]
NIPAM, Hyaluronic acid, Tetramethylethylenediamine	Radical polymerization	Luteolin	Transdermal drug delivery	[62]
NIPAM, N,N′ Methylenebis(acrylamide), Polyvinyl alcohol	Precipitation polymerization	PNIPAM	Microneedle-based drug delivery	[94]
NIPAm, glycidyl methacrylate, tetrahydrofuran, 2,2-azobisisobutyronitrile,	Free radical polymerization	Ibuprofen and 5-fluorouracil	Hydrophilic and hydrophobic drug delivery	[121]
NIPAM, N,N′-Methylenebis(acrylamide), Ammonium peroxdisulfate, N,N,N′,N′-Tetramethylethylenediamine, Polyvinyl alcohol	Free radical polymerization	Doxorubicin	Cancer	[122]
NIPAM, N,N′-methylenebis (acrylamide), Acetic acid, Hydrofluoric acid, Sodium ethyl xanthate, Span 80, Tween 20, Azobisisobutyronitrile	Reversible addition–fragmentation chain transfer polymerization	Doxorubicin	Cancer	[124]
NIPAM, Tetramethylethylenediamine, Ammonium peroxdisulfate, N,N′-methylene Bis (acrylamide), Graphene oxide, Chitosan, Sodium alginate	Free radical polymerization	Graphene oxide	Wound healing	[138]
NIPAM, Graphite oxide–nano silver, Ammonium persulfate, Tetramethylethylenediamine	Free radical polymerization	Graphite oxide, nano silver	Wound healing	[139]
NIPAM, N,N,N′,N′-Tetramethylethylenediamine, N,N-Methylenebisacrylamide, Bis-acrylamide, Ammonium persulfate	Free radical polymerization	Metformin hydrochloride	Diabetic wound healing	[141]
NIPAM, HEMA, Poly(*ε*-caprolactone)	Polymerization	Recombinant human BMP-2 and VEGF	Bone regeneration	[144]
NIPAM, 2-methylene-bis-acrylamide, Hydroxyapatite	Electrochemical polymerization method	Oxacillin	Tissue engineering	[146]
NIPAM, Gelatin, N,N,N′,N′,N″-pentamethyl diethylenetriamine, N-hydroxysuccinimide	Atom transfer radical polymerization	Gelatin	Bone regeneration	[147]
NIPAM, N-acryloxysuccinimide, Poly(ethylene glycol)	Reversible addition–fragmentation chain transfer polymerization	Dexamethasone	Ocular drug delivery	[153]
NIPAM, Polyethylene glycol, N,N,N0,N0-tetramethylethylenediamine	Free radical polymerization	-	Ocular drug delivery	[155]
NIPAM, 2, 2-azobisisobutyronitrile	Bulk polymerization	-	Ocular bioadhesive	[156]
NIPAM, 2-(dimethylamino)ethyl methacrylate,	Atom transfer radical polymerization	siRNA	Sustained drug delivery	[167]
NIPAM, Adenosine triphosphate	Free radical polymerization	Magnolol (MAG) nanocrystals	Parkinson’s disease	[168]
NIPAM, Gelatin, Silk fibroin	Light-induced radical polymerization	-	3D printing of kidney tubules	[169]

**Table 2:** Patents published on PNIPAM-based smart hydrogel applications in biomedical fields (source: Espacenet.com, https://worldwide.espacenet.com/ access date 5 January 2025).

Patent No.	Title of Invention	Application	Year	Ref.
CN102580633A	Preparation method of graphene oxide/poly(N-isopropylacrylamide) composite hydrogel	Drug delivery and tissue engineering	2012	[176]
CN104825390A	Preparation method of camptothecin controlled release organic/inorganic hybrid material POSS/PNIPAM-b-PDMAEMA	Cancer	2015	[177]
CN104490489A	Method for preparing tissue engineering blood vessel based on 3D bioprinting technology	Tissue engineering	2015	[178]
CN105561334A	Temperature targeting-based nanogel gene delivery compound, and preparation method and application thereof	Cancer	2016	[179]
CN107233570A	VEGF antibody-carrying ophthalmic thermosensitive hydrogel implant and preparation method thereof	Ocular drug delivery	2017	[180]
CN108976356A	Temperature and redox-sensitive type drug delivery material connected by diselenide bonds and preparation and application thereof	Cancer	2018	[181]
CN108186607A	Preparation method of breast cancer targeted chitosan grafted polymer medicine-carrying composite material	Breast cancer	2018	[182]
CN109864968A	Amino organic silicon liposome/temperature-sensitive hydrogel composite material encapsulating water-soluble medicine	Transdermal drug delivery	2019	[183]
CN109513038A	Thermosensitive hydrogel loaded with copper metal organic skeleton nanoparticles and preparation method of thermosensitive hydrogel	Wound healing	2019	[184]
CN109528767A	Synthetic method of antibacterial preparation based on PNIPAM and silver nanometer clusters	Wound healing	2019	[185]
CN110064127A	Intelligent transdermal drug release system for controlling drug dosage of patch based on heart rate variability	Transdermal drug delivery	2019	[186]
CN110152071A	Reversible thermosensitive sealant for temporarily closing ocular trauma and syringe applied to sealant	Ocular sealant	2019	[187]
CN111533927A	Preparation method of pH and temperature dual-response UV crosslinked chitosan injectable hydrogel	Tissue engineering	2020	[188]
CN111803702A	Preparation method of cyclic gamma-polyglutamic acid modified hydrogel loaded with growth factors	Wound healing	2020	[189]
CN112999412A	Hydrogel dressing for wound healing and preparation method thereof	Wound healing	2021	[190]
KR20220043756A	Temperature-sensitive polymer-based nanocomposite capable of chemo-photothermal treatment by cancer cell targeting and near-infrared ray and method for preparing the same	Cancer	2022	[191]
US11376344B2	System for sutureless closure of scleral perforations and other ocular tissue discontinuities	Ocular sealant	2022	[192]
CN115337447A	Silver sulfadiazine hydrogel for rapid wound healing as well as preparation method and application of silver sulfadiazine hydrogel	Wound healing	2022	[193]
CN116474159A	Constant-temperature photo-thermal system and application thereof	Wound healing	2023	[194]
CN116199911A	Bi-crosslinking hydrogel and application thereof as wound healing dressing	Wound healing	2023	[195]
CN115970004A	Two-way nano-drug delivery system based on NIR-IIFL guidance and application	Cancer theranostics	2023	[196]
CN118477038A	Emodin nanosuspension temperature-sensitive gel as well as preparation method and application thereof	Wound healing	2024	[197]

## Data Availability

No new data were created or analyzed in this study.
